# Supplementary material for: Differential GTP-dependent in-vitro polymerization of recombinant Physcomitrella FtsZ proteins
Source: Sci Rep. 2025 Jan 24;15:3095. doi: 10.1038/s41598-024-85077-6 (PMC11760385; doi:10.1038/s41598-024-85077-6)
Supplement: Supplementary file 1 — Supplementary Material 1 [file 41598_2024_85077_MOESM1_ESM.docx]

# **Supplementary Information**

# **Differential GTP-dependent *in-vitro* polymerization of recombinant Physcomitrella FtsZ proteins**

Stella W. L. Milferstaedt^1,3^, Marie Joest^2,4^, Lennard L. Bohlender^1^, Sebastian N. W. Hoernstein^1^, Buğra Özdemir^1,6^, Eva L. Decker^1^, Chris van der Does^2^, Ralf Reski^1,3,4,5,*^

^1^Plant Biotechnology, Faculty of Biology, University of Freiburg, Schaenzlestr. 1, 79104 Freiburg, Germany

^2^Molecular Biology of Archaea, Faculty of Biology, University of Freiburg, Schaenzlestr. 1, 79104 Freiburg, Germany

^3^Cluster of Excellence *liv*MatS @ FIT – Freiburg Centre for Interactive Materials and Bioinspired Technologies, University of Freiburg, Georges-Köhler-Allee 105, 79110 Freiburg, Germany

^4^Spemann Graduate School of Biology and Medicine SGBM, University of Freiburg, Albertstraße 19A, 79104 Freiburg, Germany

^5^Signalling Research Centres BIOSS and CIBSS, Schaenzlestr. 18, 79104 Freiburg, Germany

^6^Present address: Euro-BioImaging Bio-Hub, EMBL, Meyerhofstraße 1, 69117 Heidelberg, Germany

*Corresponding author: ralf.reski@biologie.uni-freiburg.de

**Table S1 Oligonucleotides and the respective template accessions or templates used for the generation of the FtsZ2‑1-eGFP fusion construct.**

| **Name** | **Sequence (5‘ to 3‘)** | **Sequence Accession** |
| --- | --- | --- |
| ftsZ2-1_5HR_Fwd | CGTCTCTCTCTGTTGGGCTTGTGAGGAA | Pp3c11_17860V3 |
| ftsZ2-1_5HR_Rev | CTTGCTCACAGCTCCACCTCCACCTCCATGACGTGTCTGGCCTCGC |  |
| Kin 2-1 3'HR Rev | ACTCTCGGCATGGACGAGCTGTACAAGTAAAGGTCGTAACATTGGAATTAC |  |
| ftsZ2-1_3HR_Rev | CGTCTCAACATGTGTGATCCGTTTCG |  |
| Link_GFP_F | GGAGGTGGAGGTGGAGCTAGTAAAGGAGAAGAACTTTTCAC | U70495.1 |
| eGFP_Rev | GGCCCCAGCGGCCGCAGCAGCACCAGCCTTGTACAGCTCGTCCATGCC | U55763.1 |
| FTSZ2_1_GFP_5int_fwd | GCCACAGGGATAGTCTGGAA | Pp3c11_17860V3 |
| FTSZ2_1_GFP_5int_rev | GAACTTCAGGGTCAGCTTGC | U55763.1 |
| FTSZ2_1_GFP_3int_fwd | CGACCACTACCAGCAGAACA | U55763.1 |
| FTSZ2_1_GFP_3int_rev | GTCTCACTGCGTCTTCGTCA | Pp3c11_17860V3 |

**Table S2 List of proteins significantly interacting with Physcomitrella FtsZ2-1 as determined by Co-IP and subsequent MS analysis.**

The list can be downloaded from Zenodo (https://zenodo.org/records/10648779).

Table S3 Oligonucleotides used for Ligation Independent Cloning in this work.

Due to the cloning strategy, all forward oligonucleotides (fwd) include the sequence 5’ AGAAGGAGATATAACTATG 3’ at their 5’ end; all reverse oligonucleotides (rev) include the sequence 5’ GGAGATGGGAAGTCATTA 3’ at their 5’ end.

| **Name** | **Sequence (5' to 3')** |
| --- | --- |
| C1pLATE_optiFtsZ1-2_fwd | GCTGTTCGTGTTACCTCTCGTTGCC |
| C1optiFtsZ1-2_H_pLATE_rev | GTGGTGGTGGTGGTGGTGGTGGTGCAGGAAACCTTTACGGTTCAGACCCTG |
| C2pLATE_optieGFP_1-2_fwd | GTTTCTAAAGGTGAAGAACTGTTCACCGGTG |
| C2optiFtsZ1-2_H_pLATE_rev | GTGGTGGTGGTGGTGGTGGTGGTGCAGGAAACCTTTACGGTTCAGACCCTG |
| C5pLATE_optieGFP_2-1_fwd | GTTTCTAAAGGTGAAGAACTGTTCACCGGTG |
| C5optiFtsZ2-1_H_pLATE_rev | GTGGTGGTGGTGGTGGTGGTGGTGGTGACGGGTCTGACCACGTTTACG |
| C6pLATE_optiFtsZ2-1_fwd | TCTATGCACTCTCGTTCTTCTGTTCG |
| C6optiFtsZ2-1_H_pLATE_rev | GTGGTGGTGGTGGTGGTGGTGGTGGTGACGGGTCTGACCACGTTTA |
| C9_mKO2_optiFtsZ2-1_pLATE_fwd | AGAAGGAGATATAACTATGGTGAGTGTGATTAAACCAGAGA |
| C9_mKO2_optiFtsZ2-1_pLATE_H_rev | GGAGATGGGAAGTCATTAGTGGTGGTGGTGGTGGTGGTGGTGGTGACGGGTCTGACCACG |
| C9_mKO2_H_pLATE_fwd | AGAAGGAGATATAACTATGGTGAGTGTGATTAAACCAGAGATG |
| C9_mKO2_H_pLATE_rev | GGAGATGGGAAGTCATTAGTGGTGGTGGTGGTGGTGGTGGTGTGAATGAGCTACTGCATCTTCTACC |

Table S4 Oligonucleotides used for Gibson cloning and sequencing in this work.

| **Name** | **Sequence (5' to 3')** |
| --- | --- |
| pre3pJET_oFtsZ1-2_fwd | TCTTCCGGATGGCTCGAGTTTTTCAGCAAGATATGGTTCGTGTTACCTCTCGTTGCCGTT |
| pre3oFtsZ1-2_eGFP_rev | ACAACACCGGTGAACAGTTCTTCACCTTTAGAAACCAGGAAACCTTTACGGTTCAGACCCTG |
| pre3oFtsZ1-2_eGFP_fwd | TGTTCCGTCAGGGTCTGAACCGTAAAGGTTTCCTGGTTTCTAAAGGTGAAGAACTGTTCACCGGTG |
| pre37oeGFP_pJET_rev | TGAGAATATTGTAGGAGATCTTCTAGAAAGATTTATTTGTACAGTTCGTCCATACCCAGGGT |
| pre7pJET_oFtsZ2-1_fwd | TCTTCCGGATGGCTCGAGTTTTTCAGCAAGATATGTCTATGCACTCTCGTTCTTCTGTTCG |
| pre7oFtsZ1-2_eGFP_rev | ACAACACCGGTGAACAGTTCTTCACCTTTAGAAACGTGACGGGTCTGACCACGTTTA |
| pre7oFtsZ1-2_eGFP_fwd | CGTCTTTCCTGCGTAAACGTGGTCAGACCCGTCACGTTTCTAAAGGTGAAGAACTGTTCACCGGTG |
| pre2oeGFP_FtsZ1-2_rev | GCACCCAGCCAACGGCAACGAGAGGTAACACGAACTTTGTACAGTTCGTCCATACCCAGGGT |
| pre2oeGFP_FtsZ1-2_fwd | CTGGTATCACCCTGGGTATGGACGAACTGTACAAAGTTCGTGTTACCTCTCGTTGCCGTT |
| pre2oFtsZ1-2_pJET_rev | TGAGAATATTGTAGGAGATCTTCTAGAAAGATTTACAGGAAACCTTTACGGTTCAGACCCTG |
| pre25pJET_oeGFP_fwd | TCTTCCGGATGGCTCGAGTTTTTCAGCAAGATATGGTTTCTAAAGGTGAAGAACTGTTCACCGGTG |
| pre5oeGFP_FtsZ2-1_rev | CGCAGAGCACGAACAGAAGAACGAGAGTGCATAGATTTGTACAGTTCGTCCATACCCAGGGT |
| pre5oeGFP_FtsZ2-1_fwd | CTGGTATCACCCTGGGTATGGACGAACTGTACAAATCTATGCACTCTCGTTCTTCTGTTCG |
| pre5oFtsZ2-1_pJET_rev | CTGAGAATATTGTAGGAGATCTTCTAGAAAGATTTAGTGACGGGTCTGACCACGTTTA |
| SUMO_FtsZ1-2_fwd | GAAGCGCATCGCGAACAGATCGGTGGTATGGCTAACCTGTCTGGTGCTAAAATCAAAGTTATCGG |
| SUMO_FtsZ1-2_rev | CTCACTGCCCGCTTTCCAGTCGGGTCGACGGTTTACAGGAAACCTTTACGGTTCAGACCC |
| SUMO_FtsZ2-1_fwd | GAAGCGCATCGCGAACAGATCGGTGGTATGTCTCTGCGTCGTATCGACCGTGCTC |
| SUMO_FtsZ2-1_rev | CTCACTGCCCGCTTTCCAGTCGGGTCGACGGTTTAGTGACGGGTCTGACCACGTTTACGC |
| pre9_pJET_mKO2 _ oFtsZ2-1_fwd | GGATGGCTCGAGTTTTTCAGCAAGATATCATGGTGAGTGTGATTAAACCAGAGATG |
| pre9_pJET_mKO2 _ oFtsZ2-1_rev | CAGAAGAACGAGAGTGCATAGATGAATGAGCTACTGCATCTTCTACC |
| pre9oFtsZ2-1_mKO2_pJET_fwd | GTAGAAGATGCAGTAGCTCATTCATCTATGCACTCTCGTTCTTCTGTTC |
| pre9oFtsZ2-1_mKO2_pJET_rev | GTAGGAGATCTTCTAGAAAGATATCCTAGTGACGGGTCTGACCACG |
| mKO2_FtsZ21_eGFP_FtsZ12_fwd | GCAGATTGTACTGAGAGTGCACCAAATTGTGAGCGGATAACAATTTGAGCTC |
| mKO2_FtsZ21_eGFP_FtsZ12_rev | CTGTGCGGTATTTCACACCGCATACAAAAAACCCCTCAAGACCCGTT |
| SUMO_fwd | GCGTAGAGGATCGAGATCTCGATCCCG |
| T7_rev | GGGGTGCCTAATGAGTGAGCTAACTCAC |

**
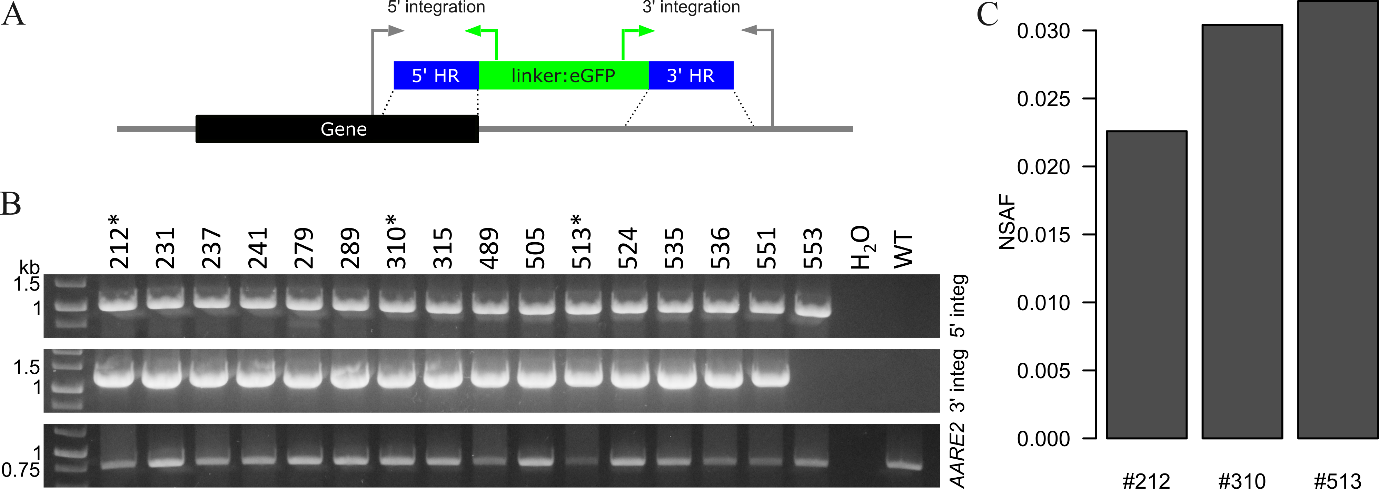
**

**Figure S1 Identification of FtsZ2‑1‑GFP fusion lines *via* PCR and Co-immunoprecipitation (Co-IP).**

Primers were designed to represent correct positioning of the knock-in construct at the desired genomic locus (Pp3c11_17860V3). (**A**) Scheme representing the positioning of the knock-in construct and the primers used for the screening PCR. (**B**) Results of the screening PCR. Expected amplicon sizes: 5’ integration: 1064 bp; 3’ integration: 1302 bp; AARE2 (Pp3c12_21080V3): 768 bp. Stars (*) indicate candidate lines that were selected for a first test Co-IP. Uncropped gel images are available from Supplemental Figure S10. (**C**) Results from the first test Co-IP with selected FtsZ2-1:eGFP fusion lines. Relative quantitative share of FtsZ2.1:eGFP in the analysed sample is represented by normalized spectral abundance factors (NSAF). A full spectrum report is available from Supplemental Table S2.


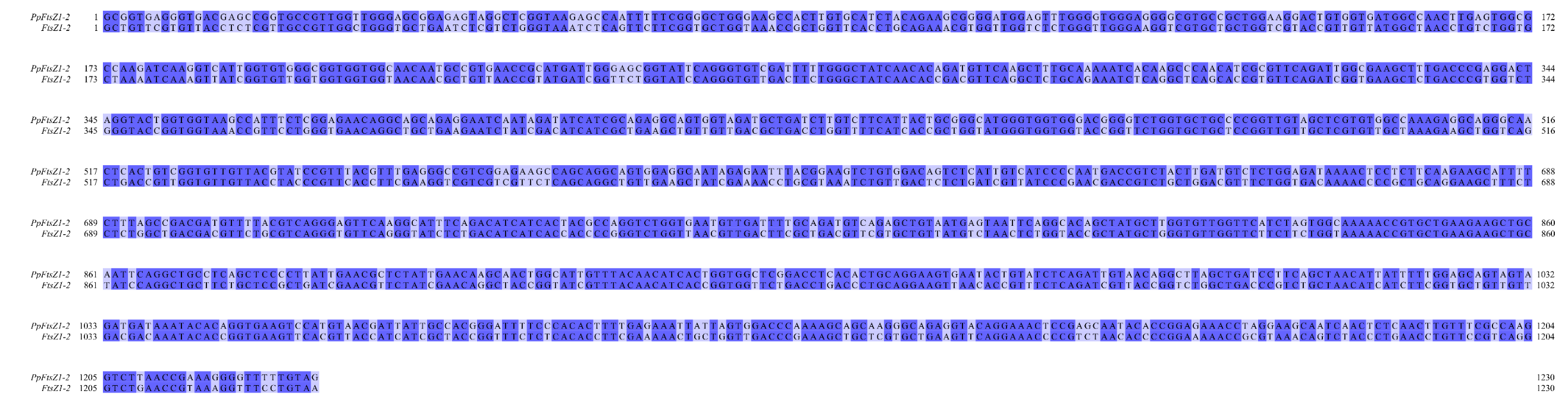


Figure S2 **Pairwise sequence alignment of Physcomitrella FtsZ1-2 (PpFtsZ1-2) and the codon optimized FtsZ1-2 (FtsZ1-2) for codon usage in *E. coli*.** Alignment was performed with Jalview (Version 2.11.2.2).


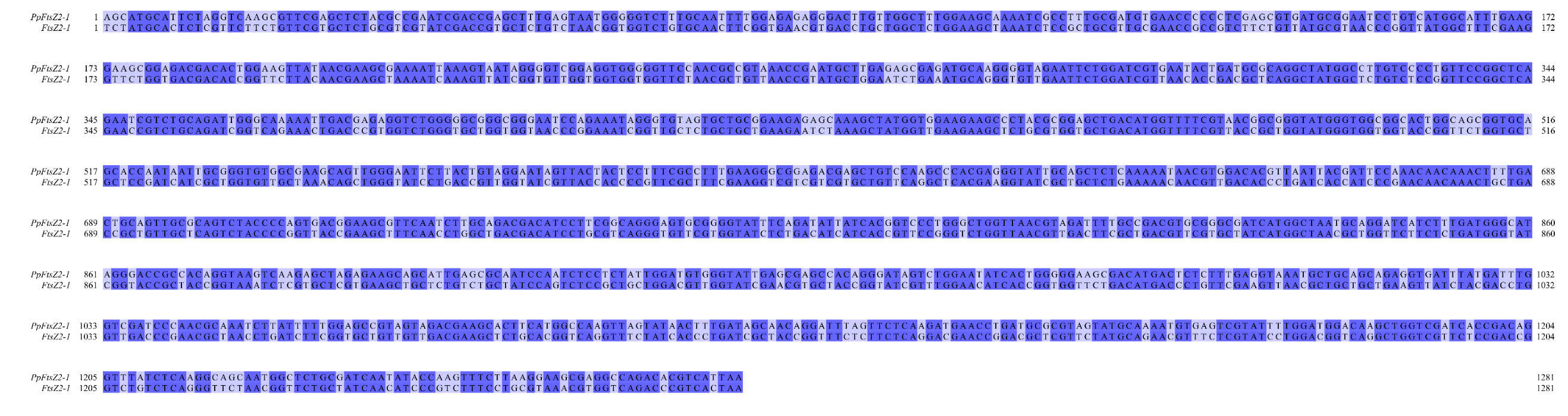


Figure S3 **Pairwise sequence alignment of Physcomitrella FtsZ2‑1 (PpFtsZ2-1) and the codon optimized FtsZ2-1 (FtsZ2‑1) for codon usage in *E. coli*.** Alignment was performed with Jalview (Version 2.11.2.2).


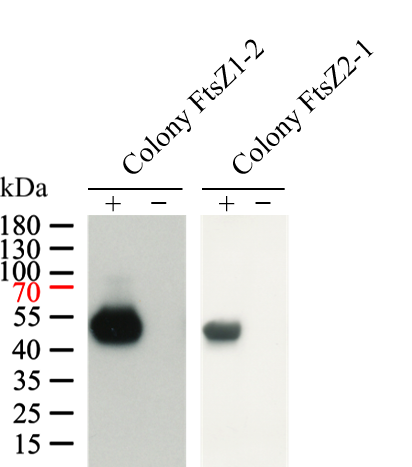


Figure S4 Immunoblot detections of protein extracts from BL21 Star™ (DE3) cells transformed with the pLATE11 vector for the expression of Physcomitrella FtsZ1-2 or FtsZ2-1.

Total protein extracts from BL21 Star™ (DE3) cells containing the respective expression constructs for the C-terminally His-tagged Physcomitrella FtsZ isoforms were separated on a 10% SDS-PAGE gel (Bio-Rad) and subsequently transferred to a polyvinylidene fluoride (PVDF) membrane (Cytiva). Immunodetection was performed using an anti-His antibody (anti-6X His tag® antibody, ab18184, Abcam; 1:1,000) and an HRP-conjugated anti-mouse secondary antibody (NA931, Cytiva; 1:50,000). Immunoblot signals were observed at the expected molecular weights, corresponding to recombinant Physcomitrella FtsZ1-2 (~44 kDa) and FtsZ2-1 (~45 kDa). The ‘+’ and ‘–’ symbols indicate whether the cells were induced or not induced with IPTG. Protein ladder: PageRuler™ Prestained Protein Ladder (Thermo Fisher Scientific). The corresponding uncropped immunodetection images can be found in Supplementary Figure S11.


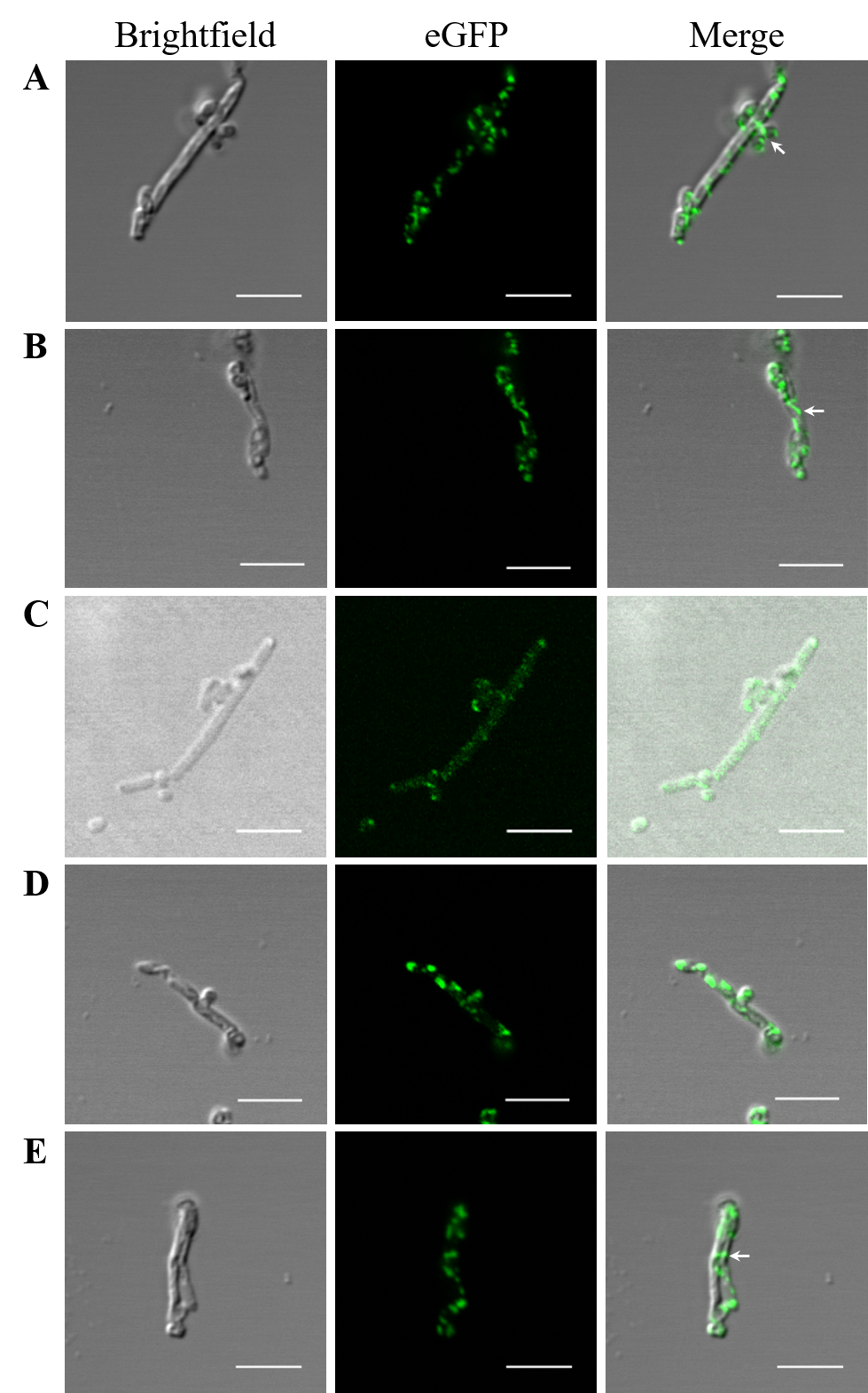


Figure S5 Confocal microscopy of eGFP-tagged Physcomitrella FtsZ1‑2 in *E. coli*.

BL21 Star™ (DE3) cells transformed with the constructs for eGFP‑FtsZ1‑2 were induced with 0.5 mM IPTG. Different elongated bacteria were observed (**A‑E**). Filament formations are highlighted with arrows. Scale bars 5 µm.


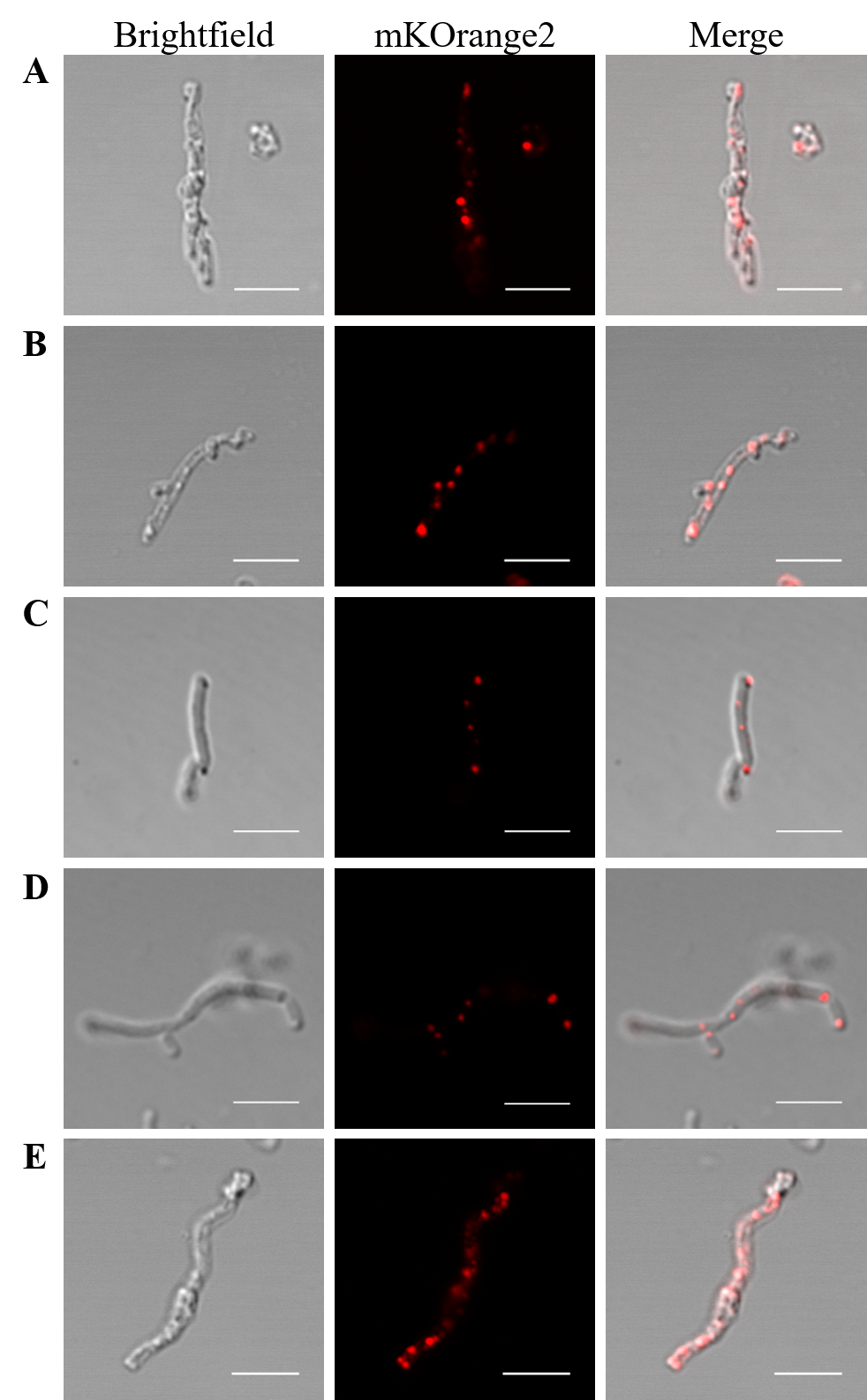


Figure S6 Confocal microscopy of mKO2‑tagged Physcomitrella FtsZ2‑1 in *E. coli*.

BL21 Star™ (DE3) cells transformed with the constructs for mKO2‑FtsZ2‑1 were induced with 0.5 mM IPTG. Different elongated bacteria were observed (**A‑E**). Scale bars 5 µm.


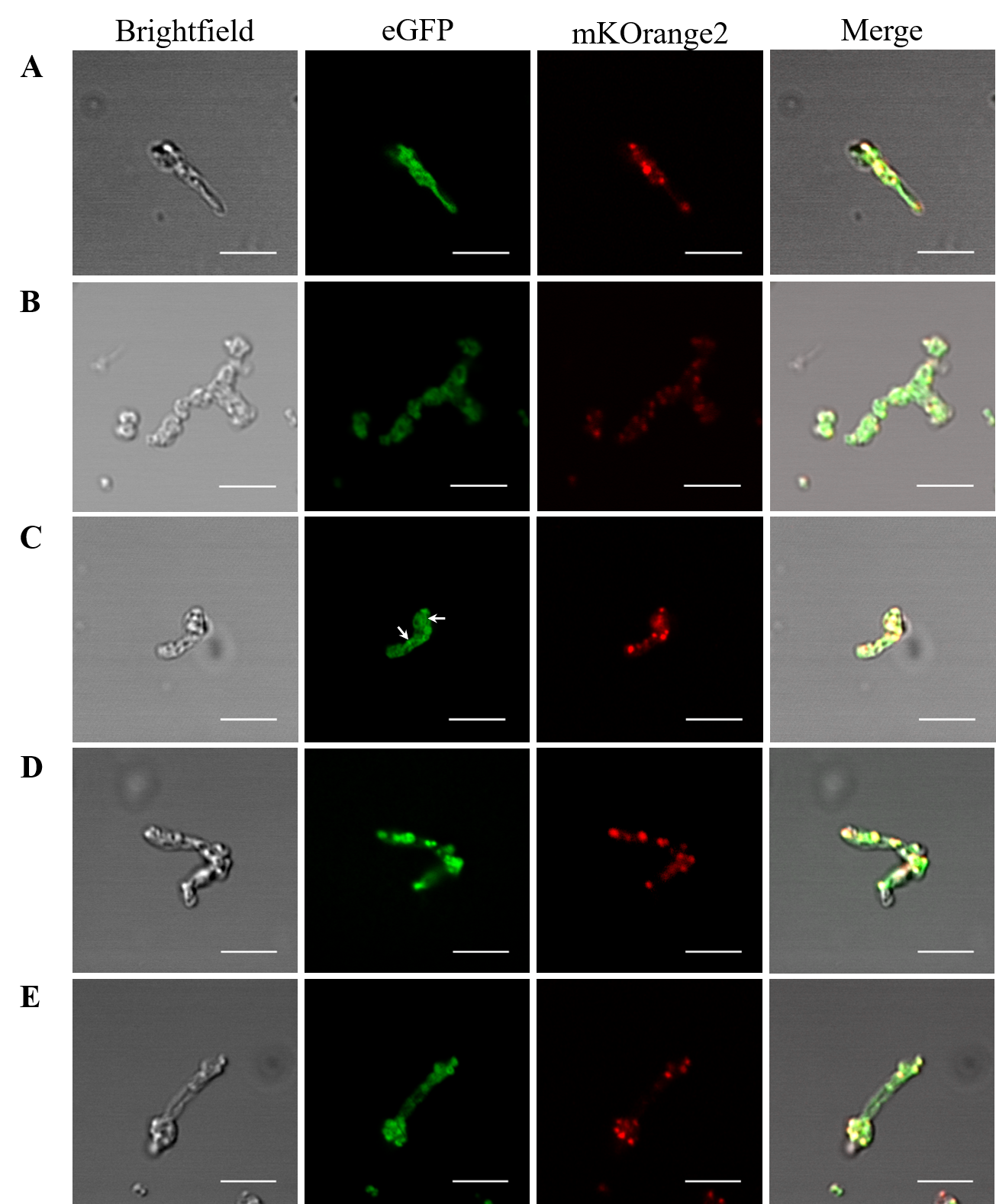


Figure S7 Confocal microscopy of eGFP-tagged Physcomitrella FtsZ1‑2 and mKO2‑tagged Physcomitrella FtsZ2‑1 in *E. coli*.

BL21 Star™ (DE3) cells transformed with the construct for eGFP‑FtsZ1‑2_mKO2‑FtsZ2‑1 were induced with 0.5 mM IPTG. Different elongated bacteria were observed (**A‑E**). Filament formations are highlighted with arrows. Scale bars 5 µm.


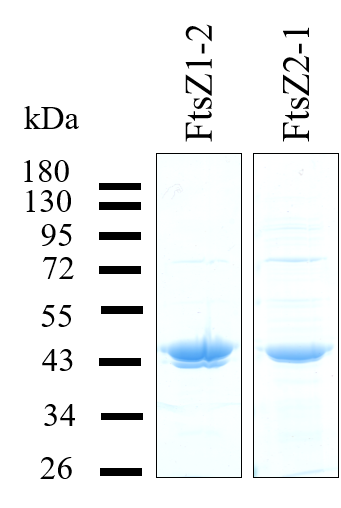


Figure S8 Coomassie-stained SDS-PAGE of purified FtsZ1-2 and FtsZ2-1.

Physcomitrella FtsZ1-2 and FtsZ2-1 were overexpressed in *E. coli* as His₆-SUMO fusion proteins. The proteins were purified using Ni-affinity chromatography, followed by removal of the His₆-SUMO tag and further purification *via* size exclusion chromatography. The fractions obtained after size exclusion chromatography are displayed. The corresponding uncropped SDS-PAGE gel image can be found in Supplementary Figure S12.


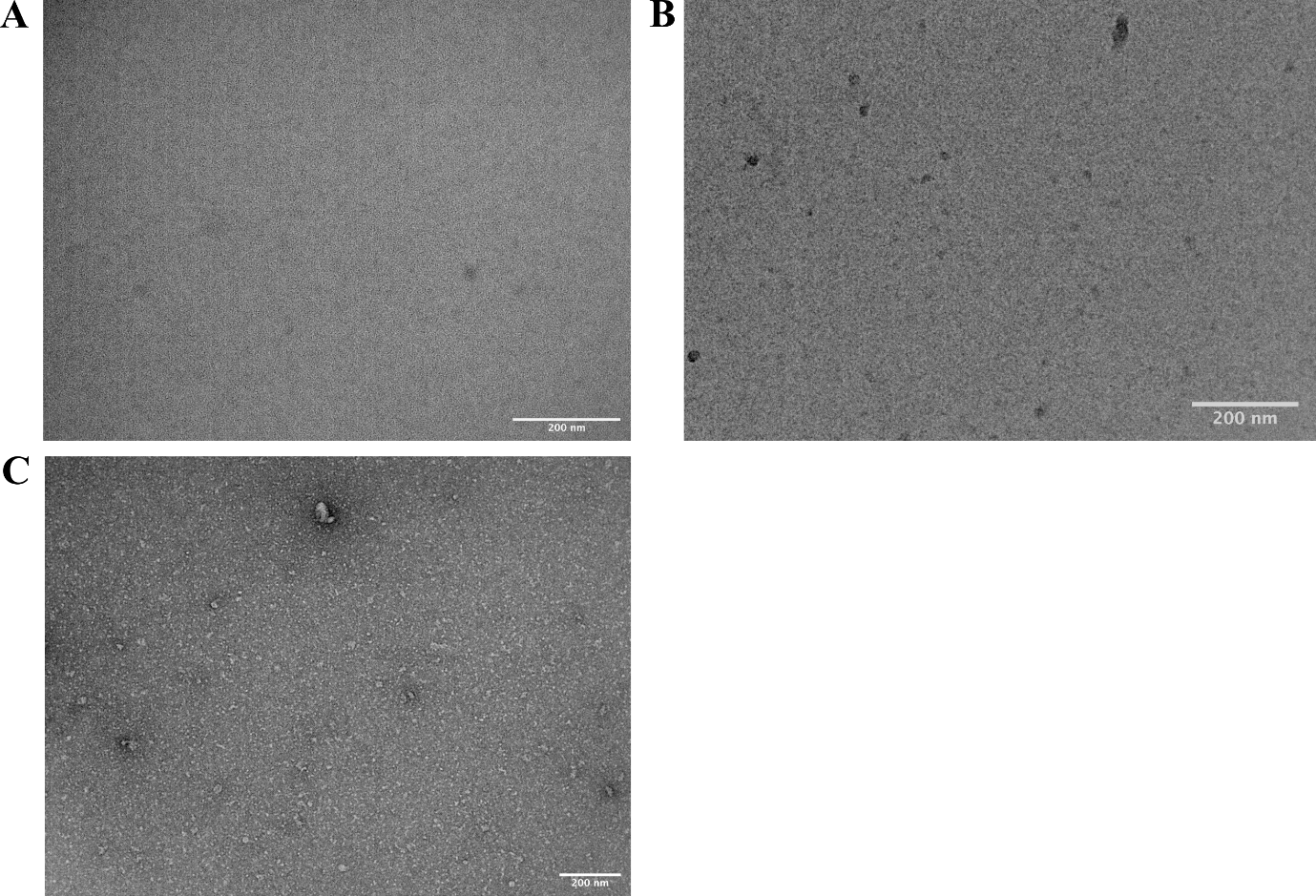


Figure S9 Negative stain transmission electron microscopy of Physcomitrella FtsZ1-2, Physcomitrella FtsZ2-1, and Physcomitrella FtsZ1‑2 together with Physcomitrella FtsZ2‑1 without GTP as negative control.

15 µM of FtsZ1‑2 (**A**), 15 µM FtsZ2‑1 (**B**) and 7.5 µM FtsZ1‑2 mixed with 7.5 µM FtsZ2‑1 (**C**) were incubated at room temperature for 5 min without GTP and then imaged by transmission electron microscopy. Representative images are shown. Scale bars as indicated. Experiments were repeated independently twice with similar results.


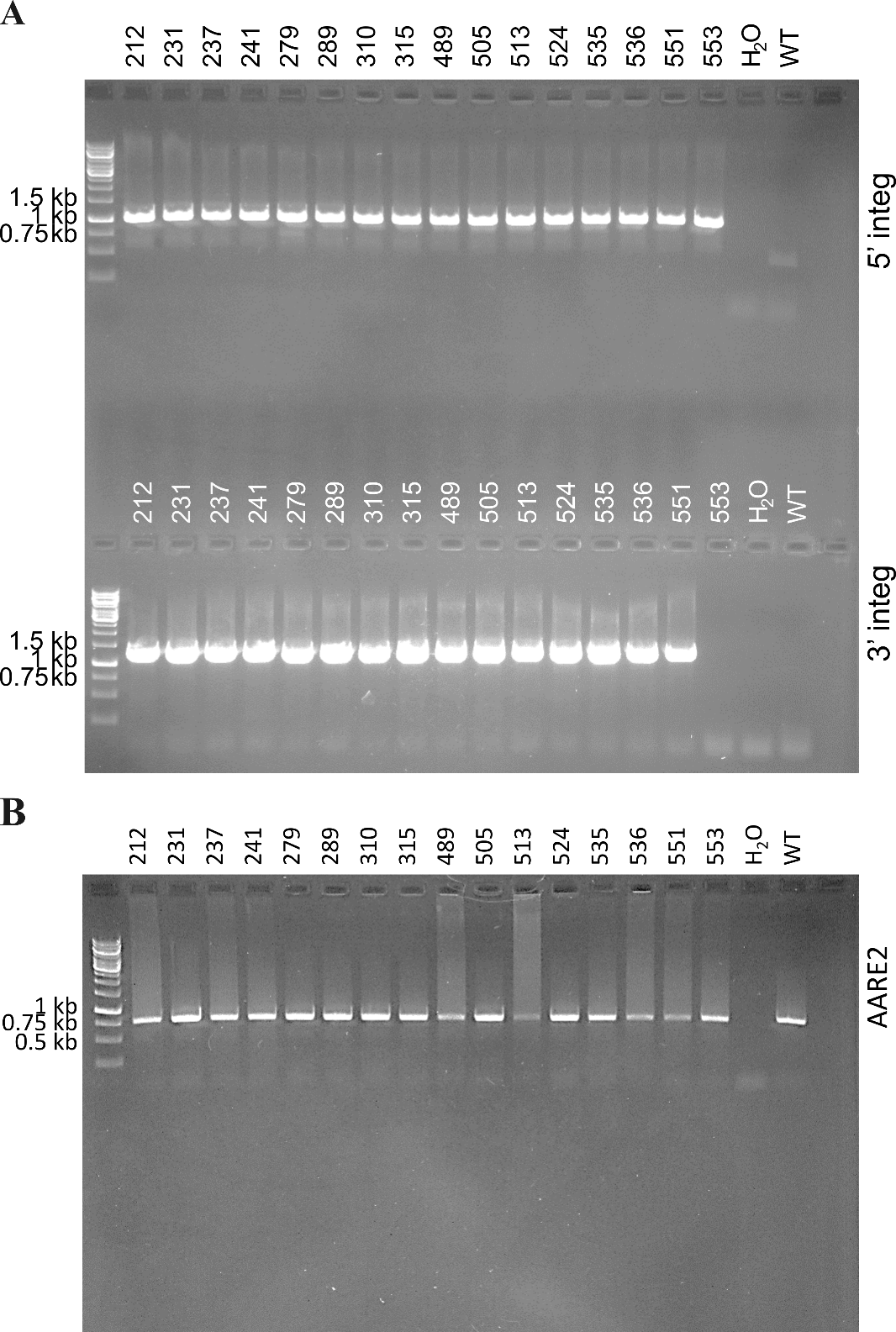


**Figure S10 Uncropped gel image of the PCR result of the screening for transgenic FtsZ2-1_GFP lines.**

(**A**) PCR testing correct integration at the selected 5’ and 3’ region. Expected amplicon sizes were 1064 bp (5’ integration) and 1302 bp (3’ integration). (**B**) Control PCR on AARE2 (Pp3c12_21080V3). Expected amplicon size was 768 bp. Primers for AARE2 were taken from [79].


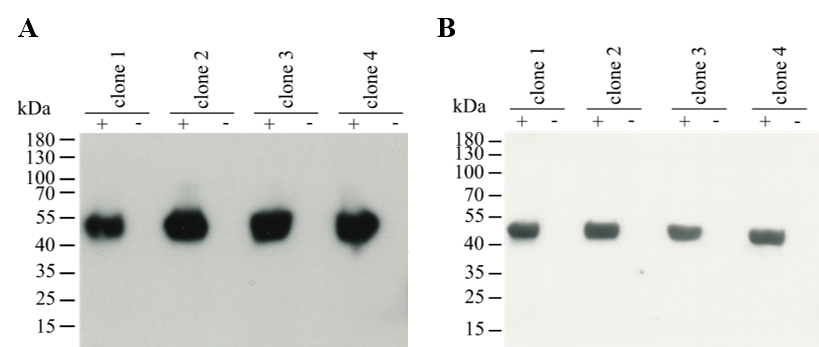


**Figure S11 Uncropped immunoblot detections of protein extracts from BL21 Star™ (DE3) clones transformed with the pLATE11 vector for the expression of Physcomitrella FtsZ1-2 or FtsZ2-1.**

Total protein extracts from four BL21 Star™ (DE3) clones containing the expression construct for either FtsZ1-2 (A) or FtsZ2-1 (B), both with a C-terminal His-tag were separated on a 10% SDS-PAGE gel (Bio-Rad) and subsequently transferred to a polyvinylidene fluoride (PVDF) membrane (Cytiva). Immunodetection was performed using an anti-His antibody (anti-6X His tag® antibody, ab18184, Abcam; 1:1,000) and an HRP-conjugated anti-mouse secondary antibody (NA931, Cytiva; 1:50,000). Immunoblot signals were observed at the expected molecular weights, corresponding to recombinant Physcomitrella FtsZ1-2 (~44 kDa) and FtsZ2-1 (~45 kDa). The ‘+’ and ‘–’ symbols indicate whether the cells were induced or not induced with IPTG. Protein ladder: PageRuler™ Prestained Protein Ladder (Thermo Fisher Scientific). For the Supplementary Figure S4, the immunoblot detections of the FtsZ1-2-expressing clone 2 and the FtsZ2-1-expressing clone 1 were chosen.


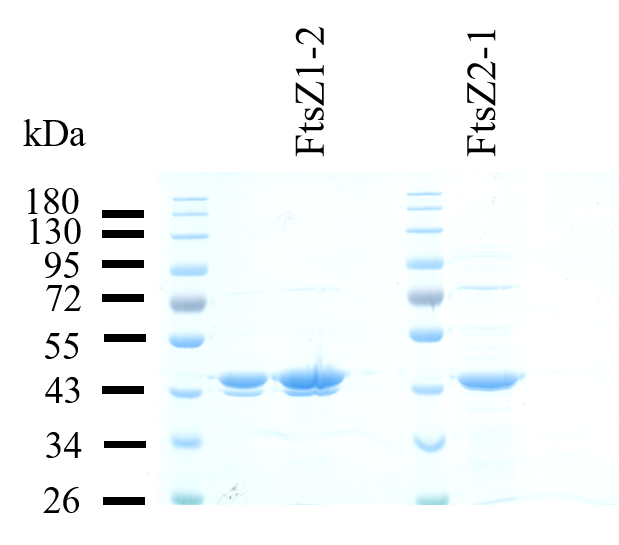


**Figure S12 Uncropped Coomassie-stained SDS-PAGE of purified FtsZ1-2 and FtsZ2-1.**

Physcomitrella FtsZ1-2 and FtsZ2-1 were overexpressed in *E. coli* as His₆-SUMO fusion proteins. The proteins were purified using Ni-affinity chromatography, followed by removal of the His₆-SUMO tag and further purification *via* size exclusion chromatography. The fractions obtained after size exclusion chromatography are displayed.
